# Pathophysiology of Demineralization, Part I: *Attrition, Erosion, Abfraction, and Noncarious Cervical Lesions*

**DOI:** 10.1007/s11914-022-00722-1

**Published:** 2022-02-07

**Authors:** W. Eugene Roberts, Jonathan E. Mangum, Paul M. Schneider

**Affiliations:** 1 Indiana University & Purdue University at Indianapolis, 8260 Skipjack Drive, Indianapolis, IN 46236 USA; 2 Department of Biochemistry and Pharmacology, Dentistry and Health Sciences, University of Melbourne, Corner Grattan Street and Royal Parade, Parkville, Victoria 3010 Australia; 3 Melbourne Dental School, University of Melbourne, 720 Swanston St, Melbourne, Victoria 3010 Australia

**Keywords:** Fluoride, Biomechanics, Percolation, Remineralization, Hydroxyapatitie, Enamel

## Abstract

**Purpose of the Review:**

Compare pathophysiology for infectious and noninfectious demineralization disease relative to mineral maintenance, physiologic fluoride levels, and mechanical degradation.

**Recent Findings:**

Environmental acidity, biomechanics, and intercrystalline percolation of endemic fluoride regulate resistance to demineralization relative to osteopenia, noncarious cervical lesions, and dental caries.

**Summary:**

Demineralization is the most prevalent chronic disease in the world: osteoporosis (OP) >10%, dental caries ~100%. OP is severely debilitating while caries is potentially fatal. Mineralized tissues have a common physiology: cell-mediated apposition, protein matrix, fluid logistics (blood, saliva), intercrystalline ion percolation, cyclic demineralization/remineralization, and acid-based degradation (microbes, clastic cells). Etiology of demineralization involves fluid percolation, metabolism, homeostasis, biomechanics, mechanical wear (attrition or abrasion), and biofilm-related infections. Bone mineral density measurement assesses skeletal mass. Attrition, abrasion, erosion, and abfraction are diagnosed visually, but invisible subsurface caries <400μm cannot be detected. Controlling demineralization at all levels is an important horizon for cost-effective wellness worldwide.

## Introduction

**Fig. 1:**
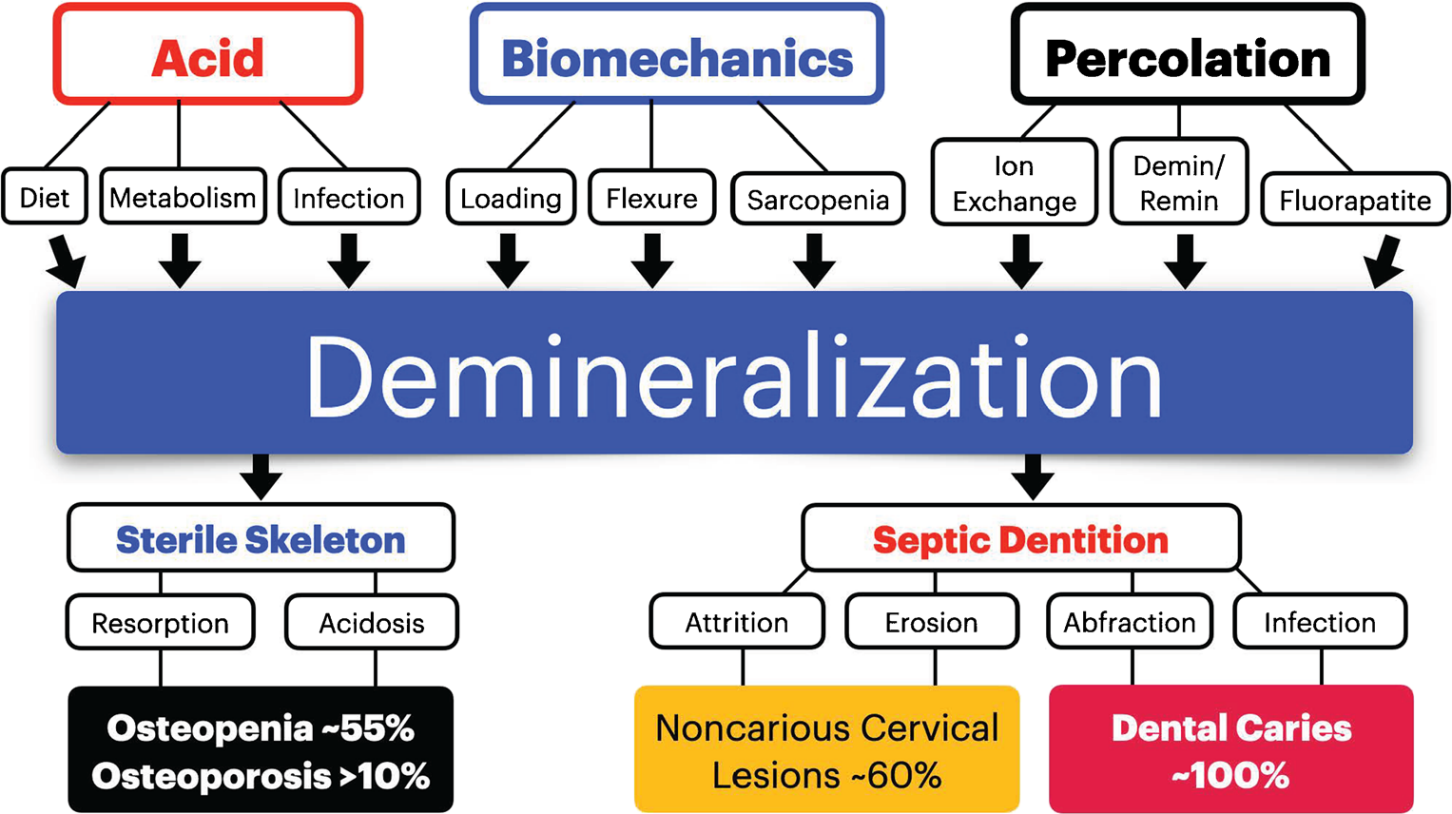
The upper aspect of the flow chart defines the etiology of demineralization as a combination of acid, biomechanics, and percolation of intercrystalline fluid. Demineralization is defined as a unique disease process. There are differential effects on the sterile bone and septic dentition compartments. Loss of mineralized tissue (osteopenia, noncarious cervical lesions, and caries) is collectively the most pandemic disease in the world (prevalence ~100%). See text for details

Demineralization of mineralized tissues has a common pathophysiology which may be infectious or noninfectious (Fig. 1). Part I of this review introduces integrated concepts for noninfectious demineralization with an emphasis on common clinical disorders. Part II discusses the superimposed variable of infection. Teeth and jaws have long been appreciated as the most heavily loaded mineralized tissues in the body [1]. The mandible is a cantilever exposed to high levels of bending and torsion [2, 3], so the remodeling (turnover) rate to repair microdamage is ~44%/year which is three times higher than for the femur [4••]. Demineralization is a disorder of structure and function [5] that is common to all mineralized tissues. The etiology involves intercrystalline fluid flow [6], metabolism [7], homeostasis [8], biomechanics [9], mechanical wear (attrition or abrasion) [10, 11], and/or biofilm-related infections [12]. Hydroxyapatite (HA) of nonliving enamel as well as the living mineralized tissues (dentin, cementum, bone) is maintained by ion exchange via percolation. Dental modeling and remodeling can occur to a limited degree, but only bone is continuously turned over with apposition and resorption [13–17]. The mineral component of all mineralized tissues evolves over time by ion exchange via fluids percolating through the microporosity of hard tissue [6, 18, 19]. An internal decrease in the mass of mineralized tissue may be reversible, but external degradation of teeth is irreversible [19]. Osteopenia and osteoporosis (OP) have a prevalence of ~55% and >10% respectively so low skeletal mass is the most common form of bone demineralization [20, 21]. Dental demineralization disorders have a much higher prevalence: 85–100% worldwide, and almost everyone (~100%) is affected over a lifetime (Fig. 1) [22–27]. Loss of mineralized tissue has profound clinical manifestations such as relatively atraumatic fractures and pain [21, 28], as well as compromises in dentofacial esthetics, function, and well-being [22–26].

Dentistry and medicine are closely related disciplines; regular dental care is directly related to overall health and wellness [29]. Demineralization disorders affect all mineralized tissues. The pathogenesis is variable but typically involves some form of acidic demineralization: environment, clastic cells or microbes [12, 14–16]. Biomechanics is essential physiology for mineralized tissue, but excessive or inadequate loading may be pathologic. OP is a metabolic problem often associated with insufficient weight-bearing exercise [30]. Occlusal wear facets are manifestations of mechanical attrition associated with a gritty and/or acidic diet as well as parafunctional habits (chronic clenching or bruxism) [31]. Bruxism (grinding) results in severe attrition of teeth, but chronic clenching (static loading) is associated with tooth fractures as well as temporomandibular joint (TMJ) degeneration [32]. Dental erosion, abrasion, and abfraction often have a dual etiology involving acidic demineralization and mechanical flexure [25–27].

Caries is a chronic bacterial infection due primarily to *Streptococcus mutans* [33–36]. It is communicable; the sterile mouth of a newborn child is inoculated with the virulent cariogenic bacteria. Dental caries is by far the most prevalent human disease in the world resulting in an annual loss of worldwide productivity of more than USD$27 billion [23], and the overall burden is increasingly among adults [22, 24]. In addition, caries is a major detriment to military readiness [34] that may be most efficiently managed with remineralization procedures prior and during deployment [35]. Because it is primarily an infectious demineralization disorder, the pathogenesis of caries is discussed in detail in part II of this review. However, the interactive pathophysiology of carious and noncarious lesions is relevant for part I.

Mineralized tissues are relatively rigid elements capable of resisting environmental challenges. Health and disease in the oral cavity are related to the specific development and morphology of craniofacial hard structures. Oral demineralization may result in substantial health problems. Emphasis on prevention and early treatment is the most expedient approach [36]. Enlightened management of dental disease requires a careful consideration of the similarities and differences between the dentition and the skeleton [37]. A tooth is a hybrid structure, septic crown with a sterile root, that is designed for heavy function. Enamel is a *nonvital* mineralized tissue with optimal properties to withstand the challenging oral environment [38]. In contrast, bones, dentin, and cementum are *vital* biologic tissues formed by osteoblasts, odontoblasts, and cementoblasts, respectively. These anabolic cells extend living processes into adjacent mineralized tissues composed of collagen matrices [18, 19, 39]. Noncollagenous proteins such as osteopontin [40] and extracellular matrix proteins [41] regulate mineralization. HA is a crystalline form of calcium (CA^++^), hydroxyl (OH^−^), and phosphate ions (PO_4_^−3^). It is the biologic mineral configuration for bones, teeth, and skin, but it is rarely encountered in geologic structures (natural rocks) [42]. Organic matrices for teeth and bones are specifically cross-liked and configured to accommodate the nucleation and crystal growth of HA crystals. Examination of the metabolism and pathophysiology of mineralized structures reveals similarities relative to acid resistance, mechanical loading, and susceptibility to infection [19, 25, 26, 43, 44].

## Developmental Morphology

**Fig. 2:**
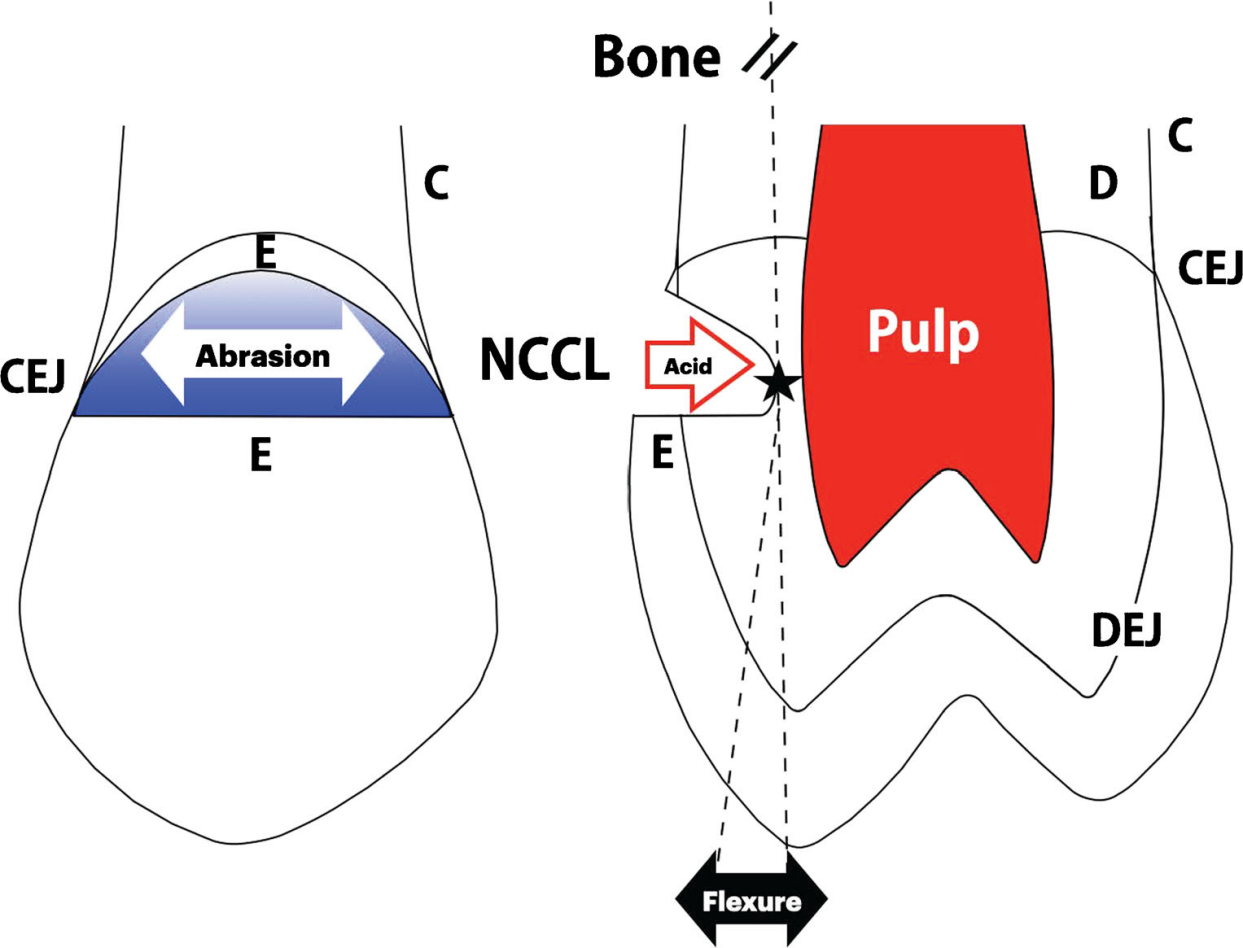
Labial (left) and cross-sectional (right) planes of a maxillary premolar show dental structures: enamel (E), dentin (D), pulp, cementum (C), dentino-enamel junction (DEJ), and cementoenamel junction (CEJ). A noncarious cervical lesion (NCCL) is depicted in both planes. The multifactorial etiology for a NCCL is (1) toothbrush abrasion, (2) dietary acid, and (3) functional flexure. The line of force for non-axial and lateral loads (dotted lines) produce surface flexure in tension and compression that results in mechanical damage at the stress riser (star) along an enamel surface or within the maximum depth of a NCCL. See text for details

Collectively, the stomatognathic system supports three principal life support functions: breathing, mastication, and mating success [45, 46]. The maxilla and mandible are secondary bones that evolve following the embryonic patterning of the skeleton. After development of the gut, neural crest cells differentiate and migrate to the ventral surface of the embryo. They induce pharyngeal and facial mesoderm to form the specialized musculoskeletal structures of the head, pharynx, and neck [46]. Teeth develop within the jaws, erupt into the oral cavity, but remain anchored in bone. The vital root of a tooth is connected to the supporting alveolar process by a periodontal ligament (PDL), a stress-bearing, connective tissue interface that has bone modeling capability similar to periosteum [14, 37, 47]. Biomechanics, T-lymphocytes, and RANK-L control the site-specific osteoclastic resorption in the PDL similar to other osseous tissues [37, 47, 48]. Modeling is defined as a change in size or form of a bone [14] and remodeling is turnover: resorption of an internal cavity that is refilled with new bone [16, 17]. Both cell-mediated processes help adapt the functioning dentition to its supporting bone. Bone size and morphology are controlled by biomechanics: physiologic loads delivered by muscles, soft tissue posture, or applied mechanics [14, 37, 47]. Teeth can demonstrate anabolic modeling in the pulp chamber by forming reactionary or reparative dentin in response to varying forms of inflammatory stimuli, most notably dental caries [49, 50]. In addition, there are two types of catabolic modeling that involve clastic cells: cervical [27] and mechanically-mediated root resorption (Fig. 2) [51]. Both of these resorption disorders are similar to the catabolic bone modeling of osteoclasts [14]. The only cell-mediated structural turnover for teeth is secondary cementum formation to refill a root resorption cavity [37, 52]. Similar to bone, the dentition develops under sterile conditions within the maxilla and mandible. Teeth erupt through osseous tissue and mucosa resulting in the crown emerging into the septic environment of the oral cavity. In addition to dental-related support for mastication and respiration, the oral cavity participates in a complex physiology that affects systemic health at multiple levels [52].

All mineralized tissues are patterned by a unilateral genetic process, so, one side of the body is the mirror image of the other [39]. Long bones are patterned as primary events during passive embryonic development, i.e., prior to muscle development. However, the maxilla and mandible evolve as secondary structures in a dynamic environment after the muscles of mastication are formed and functioning [45, 46]. The teeth are subsequently patterned within the jaws by the dental lamina. There is little environmental influence on tooth size and shape, e.g., antimeres are nearly identical bilaterally. On the other hand, bone form, size, and density are strongly affected by biomechanics [53]. For instance, sidedness of the limbs can vary considerably according to differential loading [54]. In effect, teeth are *genetic constants,* but mature bones are *environmental variables.*

## Dental Components

The articulating crowns of teeth require a strong, dense, and stable material that is resistant to acidic attack and heavy loading. As the hardest substance in the body, enamel is a genetically distinct mineralized tissue that achieves its high density by a catabolic macromolecular mechanism [55] rather than mineralization of a collagen matrix [14, 37]. Forming dense enamel involves a unique genetic process. HA-like crystals (nanoribbons) form a matrix-like structure for initial mineralization that resorbs during maturation to enhance the density of an HA rod (prism) within its less mineralized sheath [55–57]. However, the initiation of enamel mineralization is like osteogenesis. Nucleation of the initial HA crystals form an individual prism via mineralized collagen fibers from the dentin that extend into the developing enamel [56]. Enamel formation involves unique matrix proteins: amelogenin, ameloblastin, and enamelin. In addition, proteases MMP-20 and KLK4 function during the secretory and maturation phases, respectively [57]. Amelogenins act as nanospheres (spacers) for the growth of enamel crystals (rods). To achieve maximum tissue hardness and density, the organic matrix for the developing enamel is digested and removed during the maturation process as the rods are mineralized [55–59]. Thus, enamel evolves as a living tissue that becomes nonvital with maturity.

It is a noncellular, avascular tissue that protects underlying vital tissues (dentin and pulp). Enamel is a rigid tissue that is susceptible to traumatic fracture, crack propagation, and acidic demineralization. Contamination with ions like carbonate can render it more soluble [35, 36]. If the enamel is breached or malformed, the long-term prognosis for the affected tooth is questionable [60]. Enamel surfaces are composed of dense HA rods arranged in a configuration that is a similar for all primates [61]. The hybrid *nonvital* and *vital* morphology of teeth permits function as masticatory “battering rams” that transfer heavy loads to sterile supporting bone [4, 37, 62]. Dentin is a vital tissue sandwiched between a vascularized dental pulp and nonvital enamel (Fig. 2). The dentino-enamel junction (DEJ) is the vital frontier at the terminal ends of the dentinal tubules which are extensions of the odontoblasts that line the pulp. The DEJ is a critical patterning structure that is formed early in tooth development [63, 64]. Dentin forms first and then it induces enamel at the DEJ interface. Once the enamel of the crown is patterned, dentin continues to grow in an apical direction to form the root. The mechanism involves β-catenin induction of Hertwig’s epithelial sheath to form root dentin which is subsequently covered with vital cementum [65]. The interface of enamel and root cementum at the cervical margin of the crown is the cementoenamel junction (CEJ). Enamel is well designed to resist heavy loads, thermal gradients, chemical attacks, and sustained masticatory function [56]. Heavy impact loads are subsequently transferred to supporting bone [62, 66]. Under physiologic conditions, fatigue failure (spontaneous fracture) of teeth is controlled by: (1) PDL sensitivity to limit loading, and (2) physiochemical mineral turnover (demineralization, remineralization) to maintain material integrity [19, 48, 66].

Teeth form in protected tooth buds within the jaw bones. After emerging into the oral cavity, a tooth has a limited capacity for growth as evidenced by completion of root and apex formation. Crown trauma and malformation are not physiologically repairable because enamel is a nonvital tissue. On the other hand, dentin and cementum are vital tissues, so a fractured root that is reduced and well-stabilized in an alveolus can heal much like a fractured bone via resorption of traumatized tissue, and formation of new cementum as well as dentin [49, 50, 67]. Furthermore, the pulp of traumatized tooth can revascularize to restore tooth vitality [68]. The closest parallel in bone physiology is when dead cortical bone (a sequestrum) remodels with vascularized cutting/filling cones to form vital secondary osteons [13, 16, 17].

## Comparative Physiology

In contrast to the limited growth and healing capacity of teeth [49, 50], bone is a highly adaptive mineralized tissue that grows, resorbs, remodels, adapts, and heals in a dynamic environment over a lifetime [13–17]. Osseous modeling, remodeling, and bone mass are controlled by hypothalamic, genetic, mechanical, endocrine, and inflammatory signals [37, 69–71]. Lamellar bone achieves toughness to resist fracture via a cross-lined collagen matrix that is progressively mineralized [37]. Osteoblasts produce new bone matrix that must mature for 7–10 days prior to primary mineralization which is the active deposition of about 70% of the total bone mineral. The partially mineralized new osseous tissue undergoes a physiochemical secondary mineralization for the following 6–12 months to complete the bone formation process [14, 37, 66]. Secondary mineralization increases the hardness and stiffness of bone tissue [72] via a crystal growth process that is like the internal remineralization of enamel [19, 33, 35].

Metabolic activity of the dentition pales in comparison to the skeleton. Teeth play no direct role in Ca^++^ hemostasis, but all mineralized tissues probably participate at least indirectly. Osteocytes and their canalicular processes transport Ca^++^ between the bone fluid compartment and bone lining cells. Vitamin D–dependent cell membrane transport pumps Ca^++^ out of bone lining cells into the extracellular fluid (ECF) [37, 66, 73], which supports serum Ca^++^ levels [73, 74]. Cyclic demineralization and remineralization in concert with remodeling (turnover) repair microdamage and support calcium homeostasis (Fig. 1) [73–77]. In addition, osteocytic osteolysis [75] supports serum Ca++ levels in both health and disease by promoting resorption around lacunae and canaliculi. Teeth are susceptible to age-related fatigue failure that may require clinical extraction or restoration [78]. On the other hand, bone has the potential to avoid spontaneous fracture by repairing microdamage accumulation with ongoing remodeling (turnover) [13, 16, 17, 37, 76]. Bone remodeling not only repairs microdamage but also helps to support serum Ca^++^ levels [14, 73–75]. It bears repeating that the body of the mandible has the highest cortical bone remodeling rate in the body [4••] because of extensive linear and torsional flexure [2, 3]. In addition, the mineral fraction of bone is continuously modified with cell-mediated homeostasis as well as physiochemical ion exchange [73, 74]. Ultimately, osteopenia is a metabolic bone disorder reflecting long-term negative calcium balance. However, to a certain extent low bone mass is physiologically reversible [70, 71, 73, 74]. Decreased muscle mass and grip strength (sarcopenia) are reliable indicators for revealing prevalence of osteopenia (47.8%) and osteoporosis (20.7%) in women aged 60–79years (Fig. 1) [79]. Internal demineralization of enamel is best managed by prevention and remineralization strategies [35, 80–82]. However, loss hard tissue on the surface of teeth by attrition or erosion is irreversible [83–85]. Extensive wear of the dentition is an indication for surgical and restorative procedures [62, 78].

## Percolation of Mineralized Tissue

Enamel rods are organized as linear prisms oriented perpendicular to the DEJ. In a cross-sectional view, rods are shaped like keyholes [85] and separated by the peripheral inter-rod substance that originally guided the mineralization of each prism [38]. Since enamel functions under challenging septic conditions, it must be resistant to mechanical, chemical, and microbial attack [83–87]. As a nonvital tissue, the stability of enamel depends on its dense HA structure, low solubility potential, and continuous mineral maintenance by demineralization and remineralization [19, 80–82]. Microporosity within dense enamel is permeable to water and ions but not to larger structures such as microbes, biofilm, and food. In addition, enamel is resistant to demineralization because of a high solubility product that renders it less susceptible to acidic attack [87–89].

Clinically, sound enamel appears to be a solid prism, but the HA crystals are actually separated by small intercrystalline spaces that are more prominent in the rod sheaths [81, 89–93]. Hypomineralization creates a slightly porous structure that contains increased water and organic material [89–91]. This pattern of differential mineralization is associated with changes in modulation of the enamel-forming cells (ameloblasts) during the maturation stage [59, 92]. The rod sheath mineral (inter-rod substance) is not as tightly packed so it can serve as a path for acidic demineralization [93]. This problem is best controlled by percolation of optimal levels of fluoride ions (F^−^) to form a gradient of fluorapatite (FA) that is most concentrated at the enamel surface. Fluoride-rich apatite is deemed FA. Micropores connecting the intercrystalline spaces allow enamel to function as a semi-permeable material [55, 80, 81] that restricts bacteria and large molecules, but allow the passage of water and ions, particularly Ca^++^, PO_4_^3−^, OH^−^, and F^−^. Enamel like all other mineralized tissues is exposed to continuous cycles of mineralization and demineralization [19, 33, 89]. The continuous percolation of ionic fluid through the mineral fraction provides deep remineralization of HA which may involve substitution of F^−^ for OH^−^ to produce FA^−^ [89]. This mechanism is more efficient for inter-rod substance (enamel sheath) because it has greater intercrystalline porosity [93]. Mineralized tissue fluids contain Ca^++^, PO_4_^3−^, and OH^−^ ions that are released from or deposited into the HA fraction. Like bone [6, 94], there is a constant percolation of fluids and ions through enamel and dentin into the vascularized pulp. The flow of water and ions, particularly optimal levels of F^−^, through the dental mineralized tissues plays an important role for maintaining and adapting the mineral fraction (Fig. 1) [56, 80–82]. There are no reports that fluid flow through dental tissues supports systemic metabolism, but all mineralized tissues probably contribute to mineral homeostasis directly or indirectly.

There is a posterior flow of saliva from the minor, sublingual, and parotid salivary glands through the mouth and down the pharynx into the esophagus [95]. Saliva contains buffers such as bicarbonate to help control acidity in the diet and that produced by oral bacteria [96]. Similar to the extracellular and bone fluids of osseous tissue, water, and ions from the saliva flow into and through exposed oral hard tissues. Enamel is typically the oral interface for mineralized tissue with saliva, but cementum and even dentin may be exposed in the oral cavity if there is soft tissue recession and abrasion. As teeth erupt, saliva forms a thin coating of acquired pellicle on enamel surfaces. Pellicle is a protein layer rendering enamel less susceptible to demineralization [96–98]; however, it also serves as the base for plaque which may contribute to both caries and acidic erosion in a septic environment [98]. The closest parallel in bone physiology for dental pellicle is the thin layer of unmineralized osteoid which protects new bone from surface resorption.^37^

## Fluorine

Fluorine in its ionic form F^−^ is an important physiologic variable for mineralized tissue structure and function [94, 99, 100]. Sodium fluoride (NaF) via the diet, oral administration, and/or drinking water [99] is very effective for preventing demineralization particularly when optimal levels of the ion are present in ECF at the time the outer layers of enamel are formed (say age 2–10years) [101–103]. Despite broad-based opposition to fluoridation for many years, there are no scientific risks with F^−^ use as promoted in dentistry [104, 105]. However, the accumulative dose of F^−^ including absorption spikes in ECF should not exceed 1part per million (ppm) to avoid fluorosis [106]. With respect to noncavitated white spot lesions (WSLs), F^−^ is a double-edged sword. F^−^, Ca^++^, and PO_4_^3−^ are relatively small ions that readily penetrate the intercrystalline spaces of enamel to achieve remineralization [80–82, 101–103]. A high dose of topical F^−^ may be counterproductive because it contributes to a dense (hypermineralized) surface veneer of FA that inhibits the flow of the Ca^++^ and PO_4_^3−^ through enamel [80–82]. The physiologic demineralization and remineralization cycle is an effective mechanism for introducing optimal doses of F^−^ deep into the mineral component of all mineralized tissues [80, 89, 94]. Bone achieves demineralization of fully mineralized tissue with osteocytic osteolysis [75] or subsurface channeling via osteoclasts [15]. Enamel mineral can turnover via percolation through intercrystalline micropores [97, 98] or subsurface demineralization and remineralization [19, 33]. Internal demineralization contributes to porosity of bone [20, 21] and enamel [80–82, 101–103]. Metabolism of the osseous fluid compartment is mediated by calcitropic hormones [107]. Endocrine factors are known to contribute to ameloblast function [108], osteolysis [75], and bone fluid metabolism [37, 66, 107], but there is no evidence for hormonal control of fluid perfusion through teeth.

Incorporation of F^-^ into HA is a substitution for OH^−^ [109••]. HA is a sparingly soluble material with the chemical formula Ca_10_(PO_4_)6(OH)^2^, but spaces in the crystal matrix render it susceptible to acidic attack. The relatively rigid crystal structure is bound together with electrostatic forces that increase exponentially as the distance between positive and negative ions decreases (Coulomb’s law). Since F^−^ is smaller and more electronegative than OH^−^, substituting F^−^ for OH^−^ results in tighter packing of the ions into an apatite crystal lattice. This configuration stabilizes the matrix by increasing the attraction forces between the cations and anion [109••]. Fluoride-rich apatite is deemed FA. FA is a tightly packed apatite crystal that is less susceptible to acidic attack, so it is a very important factor in prevention, pathogenesis, and treatment of dental demineralization [110–116].

The problem with optimal fluoridation of teeth and bones is the precise control of F^−^ levels in saliva and ECF. If they are too low, inadequate FA is formed, but excessive F^−^ levels, even if only transient, are toxic to mineralized tissue forming cells: osteoblasts (bone), ameloblasts (enamel), odontoblasts (dentin) [99, 106]. Optimal F^−^ in the public water supply is more effective than oral administration for an optimal F^−^ effect in all age groups [89, 99, 100]. For young children still forming teeth, fluoridated tooth paste must be carefully administered to avoid fluorosis particularly if the water supply is also fluoridated [103–106]. Adolescents and adults achieve FA protection on the outer surface and within the enamel rods via cyclic remineralization of enamel. Since everyone is exposed to some degree of natural F^−^ in the diet and water supply, it is hypothesized that FA formation via percolation is more common in the enamel sheath compared to the rod because of its naturally more porous mineral structure [93]. Optimal levels of F^−^ in saliva increases demineralization resistance of the inter-rod substance by forming a gradient of FA that progressively decreases from the enamel surface. This is an important factor in caries pathogenesis (part II), but it is also a consideration in noncarious dental erosion (Fig. 2).

Hardening of dental enamel surfaces with variations of F^−^ treatment is effective for deceasing most forms of chemical and mechanical destruction of the dentition [24, 25, 27]. In addition, aminomethacrylate copolymer has the potential to enhance the anti-erosive effect of F^−^ solutions [115]. Stannous chloride enhances the protective effect of NaF. When dentin is exposed in cervical lesions, silver-diammine-floride (SDF) and potassium-iodide (KI) harden the exposed surface and have an antibacterial effect [115, 116].

Based on excellent caries control in dentistry, F^−^ was evaluated for the treatment and prevention of OP [117–122]. Clinical trials of orally administered F^−^ increased bone mineral density in the spine, but typical OP fractures were elevated because of the decreased mechanical strength of fluorotic bone [117, 118]. It was hypothesized that oral administration of F^−^ resulted in transient peaks that produced osseous fluorosis. Devices for slow release of NaF validated the efficacy and safety of F^−^ treatment for OP when optimal levels were maintained [119]. Re-evaluation of the original negative data for F^−^ treatment is indicated [120]. The previous conclusion that F^-^ was *not* effective for treatment for OP [117, 118] was based on two experimental problems: (1) inconsistent definition for the level of osteopenia and (2) inadequate control of F^−^ levels [119]. Subsequent studies that combined oral NaF and estrogen demonstrated that mineral density is more readily controlled than the incidence of vertebral fractures [121]. Fluoride at about 1ppm in the water supply has a protective effect against OP fractures, but the fracture rate increases at levels >4ppm [122].

Overall, medicine experienced the same problems as dentistry in the use of F^−^ to prevent and treat disease. It is very difficult to control spikes in the F^−^ level from oral doses. Fluorotic bone formed under ideal conditions (<1ppm F^−^) is more resistant to bone resorption much like FA incorporation into enamel. However, even a slight increase or transient spikes in F^−^ levels may result in enamel fluorosis and weak fluorotic bone [117, 118]. Fluoride supplements mediate their actions through specific genetic signaling pathways, so a level that results in fluorotic enamel and bone in one individual may not in another [123]. All considered, the US Public Health Service decreased the recommended F^−^ concentration in water to 0.7 mg/L (or 0.7 ppm) to balance the prevention of demineralization against the risk of dental fluorosis [99].

## Biomechanics

Function, locomotion, and flexure of teeth and bones is thought to enhance fluid percolation through bones and teeth. However, exposure to repetitive mechanical loads results in accumulation of fatigue damage [76]. Mineral crystals have a limited capacity to maintain material integrity in a functional environment via fluid percolation, but the sensing mechanisms if any for mineral adaptation are unclear. Fluid percolates through enamel and optimizes physical properties by maintaining crystalline integrity of HA [124]. Formation of FA further stabilizes the mineral component and inhibits demineralization. However, the demineralization protection is probably a gradient favoring the enamel surface because formation of FA results in denser mineral that inhibits fluid flow. Dental loading induces a transient flux in the microporosity fluid which may produce mechanical signals at the dentinal interface (Fig. 2) [124]. The DEJ is a very sensitive area well known to restorative dentists because a patient must be well anesthetized to achieve pain-free operative dentistry when the DEJ is surgically penetrated. The physiologic biomechanics of enamel and DEJ function are difficult to study [125], but the mechanism can be indirectly evaluated following radiation therapy. Conventional wisdom (Frost) [16] holds that radiation-induced caries is due to decreased saliva production. However, a high level of therapeutic radiation directly damages enamel by decreasing its crystallinity and disrupting DEJ function. This compromise in normal enamel physiology contributes to radiation-induced caries [125].

## Pathologic Perspectives

Mineralized tissues are affected by a broad array of biologic, chemical, and mechanical signals (Fig. 1) [33, 102]. It is clear that teeth can be severely damaged by noncarious demineralization, but most of the fundamental research on demineralization has focused on infectious caries [104, 126••, 127••]. Progress in understanding the pathophysiology of demineralization requires removing the variable of infection. However, caries must be integrated into the discussion because it is the most common clinical disorder. For example, acidic erosion of enamel (demineralization) is a complex process involving subsurface porosity (white spots) covered with a thin residual layer of intact enamel (3–9μm thick) [127••]. These important studies indicate that demineralization is not just a progressive surface erosion, but also involves remineralization of previously decalcified matrix much like initial enamel rod formation [38]. These data document how rapidly enamel can deteriorate when coated with plaque, but also show the potential for remineralization of residual matrix when a progressive lesion is arrested. The specific pathogenesis for dental caries is outside the scope of this report, but it will be discussed in detail in part II. However, a review of caries research is pivotal for defining the mechanism of demineralization, particularly via acidic erosion.

Loading is directly related to increased bone mass, enhanced repair of microdamage, and demineralization in a sterile environment (Fig. 1); however, inadequate loading (disuse atrophy) results in a loss of bone mass by osteoclastic resorption [4, 14, 37, 39]. Since teeth do not have the turnover capability of osseous tissue [16, 17], they are susceptible to fatigue damage, i.e., cracks and fractures due to the mechanical loading of occlusion and parafunction. Flexural loading of teeth may create surface cracks that enhance demineralization particularly in stress risers at the base of cervical lesions (Fig. 2). Acidic demineralization is the mediator of mineralized tissue loss by either erosion or cell-mediated resorption [13, 16, 17, 24, 25]. Cellular (clastic) resorption within the oral cavity is rare, but it does occur with trauma-related cervical resorption near the gingival margin [112]. These often extensive lesions may be mistaken for root caries in a clinical or radiographic evaluation [112, 128]. The etiology of cervical resorption is probably an immunologic response to injury [129] rather than erosion, abfraction, or caries [35, 36, 104, 127••]. Teeth may appear discolored for many reasons including white spot formation, yellowing of enamel, root caries, cervical erosion, pulp necrosis, and/or the extensive secondary dentin formation with aging [104, 111, 128, 130].

A unique developmental process for enamel produces the most dense calcified tissue in the body: ~96% inorganic material (HA) and 4% organic material and water by weight [55–58]. In comparison, osseous tissue with the greatest mineral density is a nonvital sequestrum [131] and the abnormal bone of osteogenesis imperfecta (OI), i.e., brittle bone disease [132]. Medication-related oral necrosis of the jaw (MRONJ) results in exposed oral bone sequestra [131]. Under the influence of resorption-suppressing medications, e.g., bisphosphonates and denosumab, large sections of oral bone may die and hypermineralize to form a sequestrum particularly in areas of osseous infection [128]. Subsequently, compromised mucosa covering the nonvital sequestrum atrophies because there is no vascular supply traversing the dead bone, thereby exposing an MRONJ lesion in the oral cavity. Accelerated mineral deposition to form enamel, bone sequestrum, and OI bone may involve substantial incorporation of trace elements (Sr, Zn, and Cu) which affects mechanical properties [133]. However, hypermineralized sequestra [131] and OI bone [132, 134] are still at least 50% mineralized collagen so they are not nearly as hard or resorption resistant as enamel. Indeed, enamel is a unique genetic tissue that has no peer among other mineralized tissues relative to density, strength, rigidity, and hardness [38, 55–58].

## Demineralization

The term is defined as leaching of Ca^++^ and PO_4_^−3^ ions from the investing or supporting matrix of a mineralized tissue. Plaque often collects near gingival margins, so the cervical region of a crown is threatened by acid related demineralization as well as noncarious ablation effects due to lateral or non-axial dental loading (Fig. 2). The rod sheath, or inter-rod substance that connects each rod to adjacent prisms, plays an important role in enamel formation, maintenance, and remineralization [38, 127••]. The interprismatic substance also mineralizes, but not as densely as the adjacent enamel rods. Although an enamel surface appears to be a solid structure, there is microporosity particularly in the inter-rod substance which is susceptible to preferential FA remineralization and yellow staining [130].

All dental mineralized tissues exposed in the oral cavity are susceptible to demineralization. Loss of mass is due to attrition, abfraction, erosion, or abrasion and/or caries [22, 25, 135, 136]. Under either sterile or septic conditions, the disease process involves both chemical and mechanical factors (Figs. 1 and 2). Bones respond to mechanical loading and metabolic factors in a sterile environment. Acidosis and/or inadequate loading contributes to osteopenia [137] particularly in estrogen-deficient females [138]. On the other hand, mechanical loading enhances osseous mass but it contributes to loss of dental mineralized tissue (Fig. 1) [16, 17, 25, 76, 87, 135]. Among the mineralized tissues, enamel is most resistant to net deterioration reflecting an imbalance favoring demineralization over remineralization [33, 39, 102]. However, even enamel is susceptible to an interaction to an array of detrimental factors such as dietary acid, gastric reflux, mastication, parafunction, or bacterial infection [33, 39, 101, 104, 135].

## Mechanical Lesions

In contrast to bone surfaces controlled with cellular activity, attrition, abrasion, and abfraction affect the surfaces of teeth. Abrasion is tooth wear in a septic (oral) environment. Degeneration is a pathophysiologic feature that elicits varying signs and symptoms. Bone attrition in the knee is usually quite painful as evidenced by the clinical course for osteoarthritis (OI) [11]. On the other hand, dental attrition and TMJ degeneration may be debilitating, but the loss of mineralized tissue in the jaws rarely results in physical pain [32, 45]. Parafunction (clenching and/or bruxism) is often a clinical feature of TMJ dysfunction. Physiologic stress, anxiety, and depression are common factors in the clinical course of temporomandibular disorder (TMD) [32, 45, 139]. Excessive functional activity may result in tired or sore facial muscles, but the marked facial pain attributed to TMD is usually myofascial in origin (muscle spasms) [139, 140]. Wear of enamel *per se* does not result in pain because it is a nonvital tissue; however, tooth fracture or erosion extending to the vital DEJ and dentin may elicit sensitivity and pain [67, 68, 141]. Severe dental wear and fractured teeth are more common with parafunction of neurologic origin particularly when medication is required [142, 143]. Thus, restoration of a worn or damaged dentition due to parafunction requires strong, wear-resistant materials [78]. However, TMD management focuses on the etiology: stress, anxiety, and depression [32, 45, 139].

Mechanical wear of the dental mineralized tissues is deemed attrition or abrasion. Attrition refers to functional occlusal surfaces while abrasion is hard tissue loss on other oral surfaces (buccal or lingual). Enamel may be worn away by a gritty diet, functional occlusion, or parafunction [139–146]. On the other hand, cervical (class V) lesions are surface defects near the gingival margin. They are rarely in occlusal contact but may be sensitive lesions (Fig. 2). Cervical lesions are immediately coronal to the gingival margin and have a common prevalence of 85% with incidence of 18% [27]. They are classified according to etiology as erosion, abrasion, or abfraction [25–27]. An acidic diet particularly with low pH beverages like carbonated soda and wine demineralize enamel surfaces. Most foods including phytoliths (minute mineral particles) in plants are known to be abrasive. Superimposing mechanical factors such as toothbrush abrasion, coarse diet, and compressive flexure (abfraction) increases the prevalence of cervical lesions. The defects are usually on buccal surfaces of the dentition and are particularly common for teeth exposed to crown flexure due to heavy mastication or parafunction [25–27].

Mineral loss within enamel may be reversible, but surface attrition of teeth is a permanent loss of mineralized tissue [10, 85]. The wear of enamel is minimal with normal mastication, but bruxism and/or clenching are damaging long term [143–145]. Conventional wisdom (Frost) [16] holds that bruxism is a nocturnal habit, but that concept was not confirmed in a well-controlled sleep study [144]. Managing diurnal (daytime) parafunction requires a revision in clinical strategy [146]. Daytime clenching is also associated with clear aligner therapy [147].

Habitual, stress-related nocturnal clenching subjects the dental tissues to fatigue failure which may be manifest as split teeth, cusp fractures, and TMJ degeneration [32, 45, 148]. Bruxism results in excessive wear and it is particularly prevalent in stressed females [145]. Cracks in the outer enamel layer are common, but propagation is constrained by the radiating pattern of the rod structure [149, 150]. With normal saliva output, cracks rarely become carious. However, crack-related caries is common in patients with decreased salivary flow due to head and neck radiation [151], or methamphetamine abuse [152]. On the other hand, sustained loss of internal mineral from deep cervical lesions (Fig. 2) may result in tooth fracture, pulp inflammation, and devitalization, as well as periapical bone infection [27].

Treatment of dental attrition, abrasion, and TMJ degeneration is controversial. In the absence of profound structural damage, dental abrasion is relatively innocuous, and its management is often a patient-driven process based on self-perceived esthetics [153]. However, attrition (wear facets) and myofascial pain associated with TMD are typically managed with hard methyl methacrylate or soft ethylene-vinyl acetate orthotics. These are occlusal coverage devices commonly referred to in dentistry as “splints” but the preferred term in medicine is an orthotic (device to control movable parts). An occlusal coverage orthotic distributes the functional loads over the entire arch. The device physically protects teeth from bruxism, the most common form of dental attrition [154], but no effectiveness in managing mechanical overloads (abfraction) has been demonstrated. However, dental flexure can be controlled with a neurologic orthotic to control biting strength [59] as will be described below. Prevention and interceptive care to control the progression of cervical defects is preferred because restoration of the lesion(s) is challenging. Cervical defects affect most of the population say 60%, but the prevalence can be as high as 85% over a lifetime. Teeth demonstrating mechanical lesions should be carefully monitored to confirm the lesions are not progressing prior to restorative procedures (Fig. 2) [155, 156].

## Erosion

Erosion can potentiate the demineralization of mechanical lesions, so cervical defects are classified according to etiology [27]. Acidic foods and beverages may demineralize exposed enamel, cementum, and dentin (Fig. 2); remineralization can be accomplished with HA and F^-^ gels [157]. The acidic foods commonly associated with erosion are citrus fruits, pickles, and vinegar [158]. They may be consumed directly or as ingredients in recipes. There is a preference for acidic beverages in the western diet because they are refreshing particularly after physical exertion and they “clear the palate” for better appreciation of food. Beverages with high acid content include carbonated soda, citrus-based drinks, and wine. Natural fruit tannins are prevalent in higher quality wine which may have a pH 3.5 or less. The European culture of wine and cheese consumed together is a wise social strategy. The cheese buffers the acidity of the wine, tends to adhere to enamel surfaces, and supplies both calcium and phosphate ions for remineralization.

Overall, the dental erosive potential of the diet depends on the frequency, acidic strength, and the buffering capacity of all foods or ingredients consumed during the same meal or snack. Gastric regurgitation when associated with the frequent purging or other eating disorders may result in severe erosion particularly along the palatal surfaces of the maxillary dentition. Monitoring and treating this disorder [159–161] requires the support of psychology services. Salivary proteins and particularly hemoglobin protect against dental erosion related to gastric esophageal reflux disease (GERD) [159]. Assessing GERD damage on a regular basis is good clinical practice. Bioluminescence is a novel method for assessing patterns of demineralization on tooth surfaces exposed to erosion [161]. Stannous ions (Sn^++^) in mouth wash at 200 ppm or more help protect teeth from erosion [160].

## Combined Etiology

Mechanical flexure producing surface tension or compression may produce surface micro-cracks in the cervical region that facilitate demineralization particularly in an acidic environment [25, 135, 136]. Lateral or non-axial loads on the crown of a tooth result in flexure in the cervical area near the CEJ and soft tissue margin. This is the critical section: plane of maximal flexure in a restrained body such as a tooth firmly anchored in bone. A stress riser on a tooth produced by lateral or non-axial loads is an area where the stress is significantly greater than the surrounding region. The length of the crown relative to supporting bone usually indicates the stress riser is located on the buccal or lingual surface in the cervical area (Fig. 2). Demineralization in the cervical area is potentiated by exposure to dietary acidity. The combination of environmental acidity and moderate flexural loading produces cervical erosion that tends to be broad with relatively smooth surfaces [24, 135].

Abfraction in the cervical area of the crown occurs when occlusal forces elicit pronounced flexure in the buccal or lingual plane that is perpendicular to the long axis of the tooth (Fig. 2). From a mechanical perspective, this is the critical section (greatest cross-sectional stress) for a restrained body [162]. Mineralized tissue (enamel, dentin, cementum) at the location of the stress riser occlusal to the CEJ are exposed to compressive and tensile stresses which cause microfracture and sluffing of mineralized tissue particles. This mechanically induced demineralization process is enhanced by an acidic environment or toothbrush abrasion (Fig. 2). When abfraction is the predominate etiology, cervical ditching is *V*-shaped and progressive because the deepest part of the lesion continues to be the stress riser (Fig. 2). Cervical lesions are hygiene problems that may retain plaque, support caries, elicit a hypersensitivity reaction, and result in loss of pulp vitality [27, 163].

It is unknown if orthodontics contributes to cervical lesions associated with abfraction and abrasion, but the potential is certainly a concern. There is some indirect evidence for craniofacial anomaly patients that tooth movement contributes to tooth sensitivity and cervical lesions in some patients [164]. It is unlikely the relatively low static force (<3*N*) for orthodontic tooth movement is a direct risk because functional occlusal loads are hundreds of times greater. However, moving teeth does create transient occlusal interferences that may result in damaging tooth flexure that contributes to cervical abfraction. This mechanism of dental flexure has been described for the initiation of root resorption in genetically predisposed patients [165]. In addition, fixed appliance patients may tend to brush in a horizontal plane that produces abrasion in the cervical region (Fig. 2). Furthermore, enamel surfaces are altered when fixed appliances or aligner attachments are removed [166, 167]. It is unknown if altered enamel surfaces are predisposed to abfraction, but roughened enamel surfaces may retain plaque and facilitate caries [88]. Careful study to determine the incidence and nature of cervical lesions in orthodontic patients is indicated.

Effective management for mechanical destruction of teeth requires a thorough diagnosis and comprehensive treatment plan. Nocturnal and/or diurnal parafunction may contribute to abfraction. This problem is best controlled with a neurologic orthotic, i.e., a Hawley bite plate that opens the posterior bite slightly. It should not be worn at mealtimes to avoid extruding molars. Night wear is prescribed for nocturnal parafunction [59]. Daytime wear other than mealtime is indicated for diurnal parafunction [146]. Slight opening of posterior occlusion inhibits the polysynaptic reflex [168]^,^ thereby blocking the maximal contraction of mandibular elevator muscles. Suppressing nocturnal clenching and bruxism by inhibiting this reflex arc is effective for managing mechanical overload of the dentition manifest as TMD [169], and TMJ degeneration [32, 45]. Attrition, erosion, abrasion, abfraction are controlled by correcting the diet, use of occlusal orthotics, careful hygiene procedures, and avoiding heavy loading of the dentition [163, 168, 169].

## Noncarious Cervical Lesions

A common clinical manifestation of cervical erosion, abrasion, and/or abfraction is deemed noncarious cervical lesions (NCCLs). These unique problems in dentistry are quite prevalent (~50%) overall, but they are most common in adults (>60%) [84–87]. NCCLs are manifest as a loss of mineralized tissue along the tooth surface near the gingival margin independent of caries (Fig. 2). The etiology is abrasion, erosion, and occlusal trauma (wear facets) [86]. The etiology is variable, but flexure of teeth due to mechanical loading is a common feature of the disease process. Non-axial (lateral) loads associated with habitual clenching (parafunction) produce a surface flexure in the cervical area that exceeds the known failure stresses for enamel [87]. The maximum depth of a NCCL is a stress riser when the tooth is flexed, so abfraction tends to form and deepen a *V*-shaped lesion (Fig. 2). The focus on treatment should be controlling the etiology (parafunction) rather than restoration of the NCCLs. As previously mentioned, a neurologic orthotic (Hawley biteplate with slight posterior open bite) is effective for controlling parafunction between meals [32, 45, 168, 169].

NCCLs are a perplexing problem because a cervical lesion extending into dentin may result in tooth sensitivity [164] and pain which is difficult to differentiate from root sensitivity in adults with soft tissue recession. Furthermore, the presence of plaque in a previous NCCL may facilitate caries that infects the pulp [33, 36]. In addition, acidic lozenges, tablets, and mouth rinses may potentiate demineralization [170, 171]. Caries is a serious complication for previous NCCLs in cervical and root areas because the width of dentin is relatively thin near the cementoenamel junction (Fig. 2). An active lesion can rapidly invade the pulp, devitalize the tooth, and infect its supporting bone. Biofilm studies in bone suggest a virulence to destroy osteogenic cells and degrade osseous tissue that is independent of host immunity and osteoclastogenesis [172–175]. Thus, dental biofilm infections of periapical bone may be an increasingly serious problem.

## Conclusion

Demineralization of hard tissue involves biomechanics, metabolism, immune signaling, diet, and unhealthy lifestyle. Detrimental habits, psychologic stress, and infection can also play a role. Inadequate osseous structure is defined as osteopenia, but if it is severe and/or symptomatic, the diagnosis is OP. The etiology is typically an excessive resorption due to biomechanics (disuse atrophy) and metabolism (negative calcium balance). In the absence of dental caries, loss of tooth structure is usually attrition, abrasion, erosion, and abfraction. To effectively manage dental demineralization, diet, hygiene, and stressful lifestyle must be controlled. If the differential diagnosis indicates that parafunction is a contributing factor, a neurologic orthotic may be indicated indefinitely. Incidence and prevalence of NCCLs is a particular concern for elective dental treatment such as orthodontics.
